# Selective Leaching of Inert Mineral Product and the RO Phase in Steel Slag with Acetum to Improve Total Fe Content

**DOI:** 10.3390/ma15031242

**Published:** 2022-02-08

**Authors:** Xinkai Hou, Yiming Shi, Xiangfeng Wang, Yuyi Tang, Meng Wu, Hua Zhan

**Affiliations:** 1 College of Materials Science and Engineering, Xi’an University of Architecture and Technology, Xi’an 710055, China; shiyiming@xauat.edu.cn (Y.S.); wxf8618@xauat.edu.cn (X.W.); tangyuyi@xauat.edu.cn (Y.T.); wm1721844258@xauat.edu.cn (M.W.); keteng@xauat.edu.cn (H.Z.); 2 Ansteel Iron and Steel Research Institute, Anshan 114009, China

**Keywords:** selective leaching behavior, steel slag, inert mineral product (IMP), RO phase, acetum

## Abstract

The chemical and mineral components of the leaching residues obtained during the leaching of inert mineral product (IMP) and two samples of divalent metal oxide continuous solid solution (RO phase) by acetum at 20 °C were analyzed to reveal the selective leaching characteristics of the chemical and mineral components in steel slag, and clarify the leaching rates and differences of MgO and FeO in the RO phase. The results indicated that the content of total Fe (TFe) in the leaching residue increased, whereas the contents of CaO, SiO_2_, and MgO decreased during the leaching of the inert mineral product by acetum. Fe_3_O_4_ was insoluble in acetum. The leaching rates of the RO phase and metallic Fe were very low, while those of calcium silicate (C_2_S + C_3_S) and dicalcium ferrite (C_2_F) were quite high. MgO and FeO in the RO phase continuously leached over time, and the leaching rate of MgO reached 1.9 times that of FeO. Therefore, during the leaching of the RO phase by acetum, the FeO content increased, whereas the MgO content decreased. In conclusion, acetum leaching can effectively improve the TFe content of the RO phase and the inert mineral product.

## 1. Introduction

Steel slag is a kind of industrial solid waste produced in the steel smelting process, and its emissions are about 15~20% of the output of crude steel [1]. In 2019, China’s crude steel production was 996 million tons [2], and steel slag production was about 149 million tons, but the utilization rate of steel slag was only 29.5% [3]. Using fine steel slag powder as cement admixture and concrete admixture [4,5] is the main high added value resource utilization method for steel slag, but the promotion and application of steel slag powder is restricted due to its low hydration activity [3,6]. The conventional techniques used to improve the hydration activity of steel slag powder include carbonization treatment [7,8], high-temperature reconstruction [9,10], mechanical activation [11,12], high-temperature hydrothermal curing [13,14], and chemical activation [15,16]. Although these methods can improve the activity of steel slag powder to a certain extent, its activity is still too low to be accepted by the market. A new technique to improve the hydration activity of steel slag powder is the separation of inert minerals (i.e., RO phase, Fe_3_O_4_, and Fe) for the enhancement of the relative contents of hydration activity minerals (i.e., calcium silicate and intermediate phases) in the steel slag powder [17,18], by which the activity of steel slag powder can be considerably improved. RO phase is a continuous solid solution composed of divalent metal oxides such as MgO, FeO, and CaO [19].

The obtained inert mineral product (IMP) is rich in Fe and has potential application prospects as an Fe-producing material or dense medium for coal preparation. IMP is an Fe-rich product obtained by the pneumatic and dry and wet magnetic separation of steel slag powder. Unfortunately, the content of total Fe (TFe) in IMP is only approximately 42.0%, which fails to meet the quality requirements of the Fe ore concentrate (TFe ≥ 53%) [20]. Therefore, new technology must be developed to improve the TFe content of IMP.

Selective leaching with chemical reagents [21] is an effective method for mineral purification. Since calcium-based hydraulic minerals in steel slag are considered impurities in IMP, selective leaching of these components could effectively increase the TFe content of the latter. The price of organic acids such as formic acid is relatively high, while the inorganic acids such as phosphoric acid and sulfuric acid, if remaining in the leaching residue, will bring harmful elements such as P and S to the iron-making process. Compared with the above-mentioned acids, acetic acid is a weak, widely available, and inexpensive acid used as the preferred reagent for leaching alkaline calcium-based hydraulic impurities, and the calcium magnesium acetate produced during the leaching process is a high added value product [22]. The literature on the selective leaching of steel slag by acetic acid focuses on the leaching behavior of the chemical components of the slag and the kinetic modeling of the leaching process. Chen Lin et al. [23], for instance, concluded that leaching temperature, leaching time, and acetic acid concentration are positively correlated, whereas the solid to liquid ratio and steel slag particle size are negatively correlated with the Ca^2+^ leaching rate. Zhang Huining et al. [24] established a Drozdov-type kinetic model with a self-resistance coefficient to describe the Ca^2+^ leaching process and confirmed that leaching production solubility is not a factor influencing the leaching process. Zhang et al. [25] divided the leaching rates of Ca, Fe, Mg, Mn, Al, and Zn from steel slag observed during leaching with acetic acid solution into three levels and found that the relationships between the metal concentration in the leaching solution and the leaching temperature, liquid–solid ratio, and particle fineness follow a first-order exponential function; the authors then verified that the extraction reaction could indeed be expressed by a core shrinking model.

The existing studies have not measured the equilibrium leaching rate of ions after 3 h, and data on the leaching rate of Si are lacking. Furthermore, in the literature [23,24,25], only the leaching rate of chemical components could be obtained by measuring the ionic concentration of the solution during the leaching of steel slag by acetic acid. In addition, the leaching behavior of the mineral components of steel slag cannot be determined, the TFe content of the leaching residues cannot be measured, and the selective leaching behavior of Fe and Mg in the RO phase of the steel slag was not mentioned. Thus, further study of the leaching behavior of the chemical components of steel slag is necessary.

In this paper, IMP, as the research object, was studied to determine the chemical and mineral components of its leaching residues during acetum leaching to reveal the selective leaching characteristics of the chemical and mineral components in the steel slag. Thereafter, with the two types of RO phases as the research objects, the X-ray diffraction pattern and chemical composition of the RO phases during acetum leaching were determined to clarify the leaching rates and their differences of MgO and FeO in the RO phase. This paper aims to provide theoretical guidance for the research and development of Fe grade-improvement technology for IMP.

## 2. Materials and Methods

### 2.1. Materials

**Figure 1:**
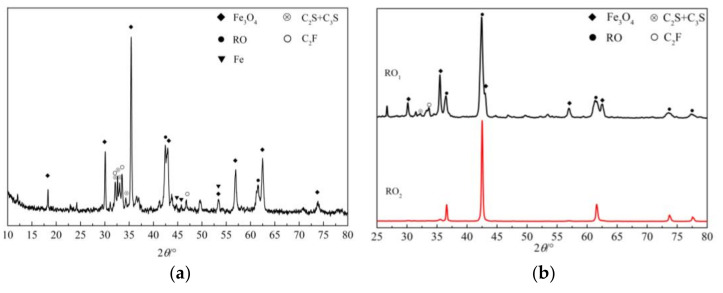
XRD patterns of three samples. (**a**) S_1_; (**b**) RO_1_ and RO_2_.

(1) IMP. This sample, marked S_1_, was obtained by the pneumatic and dry and wet magnetic separation of steel slag powder manufactured by Angang Steel Company Limited. The TFe content and specific surface area of sample S_1_ were 41.77% and 128 m^2^/kg, respectively. The mineral components of sample S_1_ were determined by XRD, as shown in Figure 1a. Sample S_1_ contained Fe_3_O_4_, RO phase, calcium silicate (C_2_S + C_3_S), dicalcium ferrite (C_2_F), and a small amount of metallic Fe.
(2) RO phase. Due to the great difference in the FeO and MgO content of RO phase in steel slag from different places of production, two RO phase samples were selected in order to comprehensively explore the dissolution characteristics of these RO phase minerals in acetic acid solution. The first RO phase sample was marked RO_1_ and obtained from steel slag powder with high RO phase content. The mineral components of sample RO_1_ were 45.8% RO phase, 30.5% Fe_3_O_4_, 15.8% C_2_F, and 7.9% calcium silicate, as determined by chemical phase analysis [26,27]. The XRD pattern of RO_1_ is shown in Figure 1b. The second sample, labeled RO_2_, was synthesized using three chemical agents at a high temperature. The XRD pattern of sample RO_2_ is shown in Figure 1b; results indicated that RO_2_ is a high-purity material containing RO phases. The chemical compositions of samples S_1_ and RO_1_ are shown in Table 1.
(3) Five analytical reagents (purity greater than 99.5%): acetic acid, methanol, KOH, sucrose, and salicylic acid. Acetic acid was used to prepare 20% acetum; KOH and sucrose were used to prepare KOSH solution; salicylic acid and methanol were used to prepare SAM solution.

### 2.2. Sample Preparation and Leaching Experiment

#### 2.2.1. Preparation of the Two RO Phase Samples

(1) RO_1_. The concentrate obtained from the dry magnetic separation of steel slag powder was wet ground for 30 min. Fe and Fe_3_O_4_ in the concentrate were removed by magnetic separation under a weak magnetic field of 70 mT to obtain tailings. Calcium silicate and C_2_F in the tailings were then dissolved by the SAM–KOSH [28] method to obtain sample RO_1_.
(2) RO_2_. Analytically pure MgO, Fe_2_O_3_, and Fe powders were used as raw materials. The powders were mixed and homogenized at an FeO:MgO molar ratio of 1, and the mixture was pressed into Φ 10 mm × 20 mm cylinders with 10 kN of force for 2 min. Sample RO_2_ was synthesized by calcining the cylinders at 1450 °C for 2 h in a tubular furnace with high-purity N_2_ as a protective atmosphere.

#### 2.2.2. Acetic Acid Leaching Experiment

The sample was ground into a powder with a particle size of less than 8 μm and put in a beaker containing 20% acetic acid solution at a solid–liquid ratio of 1 g:20 mL. The mouth of the beaker was sealed with plastic film, and the beaker was placed in a constant-temperature water bath at 20 °C. The liquid in the beaker was mechanically agitated for the required time at the speed of 300 r/min. The solution in the beaker was filtered, and the leached residue was washed with distilled water, dried at 105 °C to a constant weight, and then weighed.

### 2.3. Characterization and Calculation Methods

#### 2.3.1. Instrumentation

The chemical composition of the materials was determined by aX-ray fluorescence spectrometer (XRF, BRUKER S8 Tiger, Karlsruhe, Germany). The mineral phase components of the samples were determined using a X-ray diffractometer (XRD, D/Max2200, Tokyo, Japan). The working parameters were λ = 0.15418 nm, Cu Kα radiation source, 0.15 mm receiving slit, and 3°/min scan rate. The microscopic distribution and aggregation of typical mineral phases were observed by a field emission environment scanning electron microscope (SEM, Quattro S, Hillsborough, NC, USA). First, the sample was mixed with epoxy resin and solidified. Then, the specimen block was smoothed and polished, and its surface was sprayed with gold for 10 s for SEM observation. The operating parameters of SEM were voltage 20.00 kV, spot size 3.5, and lens distance from the sample 9.8 mm. The elemental composition of the mineral phases was determined by energy-dispersive X-ray spectroscopy (EDS, Quattro S, Hillsborough, NC, USA).

#### 2.3.2. Chemical Analysis

Determination of the CaO, MgO, and SiO_2_ contents of the samples was performed according to the standard Chinese “Methods of Chemical Analysis for Steel Slag”YB/T 140-2009 [29]. For the determination of CaO and MgO content, the samples were melted with mixed solvent firstly. The melting charge was then dissolved in hydrochloric acid to produce a 250 mL solution. Finally, 50 mL solutions were extracted and titrated with Ethylene Diamine Tetraacetic Acid (EDTA) standard solution to calculate each content according to the volume of EDTA standard solution consumed. For the determination of SiO_2_ content, the sample was melted with mixed solvent, and the melting charge was dissolved in hydrochloric acid into a solution. Then, perchloric acid was added to the solution to dehydrate the silicic acid. After filtering the solution, the precipitate was burned and weighed to obtain the mass *m*_1_. Finally, the precipitate was treated with hydrofluoric acid so that the silicon escaped in the form of silicon tetrafluoride, and the residue was burned and weighed to obtain mass *m*_2_. The difference between *m*_1_ and *m*_2_ was the mass of SiO_2_.

Determination of TFe content was conducted according to the standard Chinese “Test Method for the Content of Total Iron in Steel Slag” YB/T 148-2015 [30]. The determination principle is to melt the sample by mixed solvent, and the melting charge was dissolved into a solution with hydrochloric acid. First, add 1 g NH_4_Cl into the solution, then add ammonia to facilitate the precipitation of the Fe and Al elements in the solution. Ca and Mg in the sample can be separated by filtration solution. The precipitate was then dissolved in hydrochloric acid to make a 250 mL solution, and 100 mL solution was extracted and titrated with EDTA standard solution. The TFe content was calculated according to the volume of EDTA standard solution consumed.

Determination of the mineral phase contents of Fe_3_O_4_, metallic Fe, and the RO phase of the sample was performed as described in the literature of Hou [26]. The content of Fe_3_O_4_ is equal to that of the insoluble matter in the 10% HNO_3_ solution. The content of metallic Fe is the consumption of the solid sample dissolved in the iodide ethanol solution. The mass of the insoluble matter in the EDTA-DEA-TEA (Ethylenediaminetetraacetic acid disodium-Diethylamine-Triethanolamine) solution was determined as the total mass of Fe_3_O_4_, metallic Fe, and RO phase. The RO phase content is the total mass subtracting the above Fe_3_O_4_ and metallic Fe content.

Determination of calcium silicate and C_2_F was also conducted according to previously established methods [27]. The silicate mineral content is equal to the dissolution rate of the sample in SAM solution, and the value of the mineral phase content of C_2_F is equal to the dissolution rate of the sample in KOSH solution.

#### 2.3.3. Calculation of the FeO and MgO Contents of the RO Phase

For the RO_1_ sample and its leaching residue, the calculation method of FeO content in the RO phase is as follows. As the TFe content in the sample is equal to the algebraic sum of products of the mineral content of individual Fe-bearing and its TFe content, the content of Fe in the RO phase can be calculated from the content of minerals in the sample and the content of Fe in each mineral. The FeO content of the RO phase is equal to the Fe content of the RO phase multiplied by 1.29.

In the RO_1_ sample and its leaching residue, the calculation method of MgO content in the RO phase is as follows. Except for the RO phase, other minerals do not contain Mg or contain very little Mg, hence MgO in the sample can be regarded as from the RO phase. Therefore, the percentage of MgO in the RO phase can be calculated according to the content of MgO and the content of the RO phase in the sample.

## 3. Results and Discussion

### 3.1. Leaching of IMP

#### 3.1.1. Leaching Characteristics of the Chemical Components

**Figure 2:**
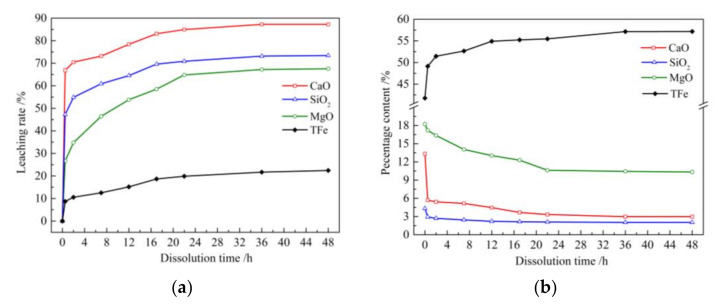
Leaching rates and contents of four chemical components of the solid material. (**a**) Leaching rates of CaO, SiO_2_, MgO, and TFe; (**b**) CaO, SiO_2_, MgO, and TFe content of the solid material.

The TFe, MgO, CaO, and SiO_2_ contents of the solid material (i.e., sample S_1_ and its leaching residues) were measured at nine specific time intervals. The leaching rate of these chemical components was calculated according to the chemical composition of sample S_1_ and the yield of its leaching residue, as shown in Figure 2.

The leaching rate curves of the four chemical components shown in Figure 2a indicate that the leaching rate of CaO remained unchanged over a duration of 36–48 h. In addition, the leaching rates of the other three chemical components changed only slightly over the same period. Thus, the leaching rates noted at 48 h were taken as the final leaching rate. The final leaching rates of CaO, SiO_2_, MgO, and Fe were 87.25%, 73.43%, 67.55%, and 22.44%, respectively. These chemical components could be divided into the following three categories according to their final leaching rates. The respective final leaching rates of CaO and SiO_2_ were 3.9 and 3.3 times higher than that of Fe. Therefore, these components were classified as high-leaching rate components. The final leaching rate of MgO was 3.0 times higher than that of Fe, and this component was classified as a medium-leaching rate component. The final leaching rate of Fe was only 25.7% that of CaO; thus, this component was classified as a low-leaching rate component.

In the literature of Zhang [25], measurement of the ionic concentrations of Ca, Fe, and Mg in the leaching solution for 3 h revealed selective extraction yields of 70–82% Ca and 10% Fe and Mg. The leaching rate of Ca was higher than those of the other elements, similar to the results in this study. However, a large difference in the leaching rates of Fe and Mg was observed between this study and the existing literature. This difference was not observed in previous studies; thus, the leaching rates of these elements were roughly classified as 10%. This may be because the sample used in the experiment in the literature was coarser and failed to fully show the leaching rate difference of Mg and Fe when processing. The leaching rate of Si was not determined in previous studies. Fe, MgO, CaO, and SiO_2_ are the main chemical components of IMP. The leaching rates of the three other chemical components were clearly higher than that of Fe. As such, these impurities could be leached by acetum to obtain leaching residues with high TFe content. Therefore, a new method to dissolve IMP with acetum to improve its TFe content can be proposed by applying the results of this study.

Figure 2a show that the leaching speed of the four chemical components is high in the early stages of leaching and then gradually decreases. The leaching process can be divided into rapid-leaching and slow-leaching stages according to leaching speed. The leaching rates of CaO and SiO_2_ initially rose sharply within 0.5 h and then rose more slowly with further increases in leaching time. Therefore, 0.5 h was considered the end of their rapid-leaching stage. Then, the leaching rates of MgO and Fe increased slowly with time and reached the inflection point of the curve at 22 h. Therefore, 22 h was considered the endpoint of their rapid-leaching stage. The longer the rapid-leaching stage, that is, the longer the time required to reach an approximately stable state, the slower the leaching rate of the samples. The respective leaching rates of CaO and SiO_2_ at 0.5 h were 76.5% and 64.4% of their final leaching rates. By comparison, the respective leaching rates of MgO and Fe at 0.5 h were 39.5% and 38.7% of their final leaching rates. Therefore, CaO and SiO_2_ had short rapid-leaching stages, and the ratio of the leaching rate at 0.5 h to the final leaching rate was great. Thus, these components may be classified as rapid-leaching components. By contrast, MgO and Fe had long rapid-leaching stages, and the ratio of their leaching rate at 0.5 h to the final leaching rate was low; thus, these components may be regarded as slow-leaching components.

A comparison of the leaching speeds of various chemical components and the influence of time on leaching speed has not been reported in the literature. Applying the results obtained thus far, the leaching time could be set according to the limit value of the content of three impurities in the leaching residues.

Figure 2b show that the contents of the four components in the leaching residues demonstrated different trends over time. TFe content increased gradually, whereas CaO, SiO_2_, and MgO contents decreased gradually over time. Similar to the leaching rates determined earlier, the content of CaO in the leaching residue remained unchanged from 36 h to 48 h, and the contents of the three other components changed only slightly over the same period. Therefore, 48 h was taken as the endpoint of the leaching process, and the chemical composition of the leaching residue at this time was taken as its final chemical composition. After the leaching of sample S_1_ by acetum, the TFe content increased by 15.40% and reached 57.17%, which met the requirements of the index [20], whereas the SiO_2_, CaO, and MgO contents decreased by 2.30%, 10.37%, and 7.89%, respectively, in the leaching residue. These components could be categorized according to the trends and magnitudes of their content changes in the leaching residue. The first category includes TFe, which showed a large increase in content; the second category includes SiO_2_, which showed a small decrease in content; and the third category includes CaO and MgO, which showed a large decrease in content.

The chemical composition of leaching residue has not been reported in the literature. In this paper, the classification of chemical components according to the variation of their content in the leaching residue not only verified the separation principle described above but also predicted the content reduction of various impurities in the leaching residue.

Similar to the leaching speed of the chemical components described above, changes in the contents of the chemical components of the leaching residue initially occurred rapidly and then more slowly. CaO and SiO_2_ contents showed rapid changes over 0.5 h. By contrast, MgO and TFe contents showed gradual changes over 22 h.

SEM-EDS can quantitatively analyze the elemental composition of a material, through which the changes of the chemical compositions of sample S_1_ before and after the leaching process can be analyzed. Sample S_1_ and the leaching residue obtained after leaching for 0.5 h (labeled S_1_-0.5) were subjected to SEM-EDS measurement. The chemical compositions of these two powder samples are listed in Table 2. Comparison of the chemical compositions of the two samples indicated that the elements could be divided into three types according to their trend and variation range. The first type includes Fe, which showed a large increase in content; the second type includes Si and Al, which showed a small decrease in content; and the third type includes Ca and Mg, which showed a large decrease in content. The SEM-EDS results were consistent with the evolution characteristics of the chemical components of the leaching residue determined by chemical analysis.

#### 3.1.2. Leaching Characteristics of the Mineral Components

(1) SEM testing

**Figure 3:**
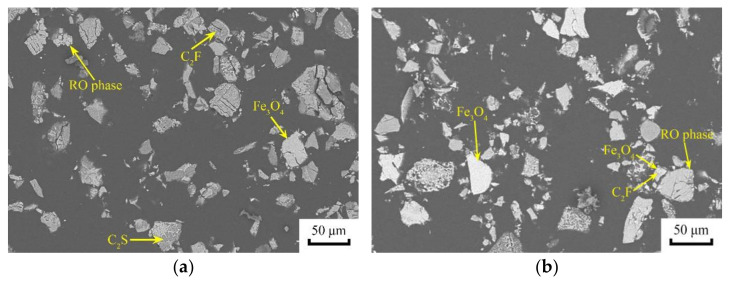
SEM-BEI images of mineral phases in sample S_1_ and sample S_1_-0.5. (**a**) sample S_1_; (**b**) sample S_1_-0.5.

Figure 3a, b show the SEM-BEI images of the mineral phases in samples S_1_ and S_1_-0.5. The mineral phases in steel slag could be divided into three types according to the contrast of the phases as determined by SEM-BEI: dark gray calcium silicate, gray C_2_F, and white inert minerals [31]. Figure 3b show that the content of calcium silicate decreased significantly, that of C_2_F changes minimally, and that of inert minerals increases significantly relative to the peaks observed in Figure 3a. Statistical analysis of the mineral phase contents from the SEM-BEI images showed that, compared with those in sample S_1_, the calcium silicate content decreased by approximately 15%, the C_2_F content increased by approximately 3%, and the inert mineral content increased by over 15% in sample S_1_-0.5.

Although petrographic quantitative analysis offers a straightforward means to determine mineral contents, ensuring the representativeness of the samples and selected fields of view is difficult, the accuracy of the determination results is low. Therefore, other mineral phase content determination methods were needed to confirm the above results.

(2) XRD analysis

**Figure 4:**
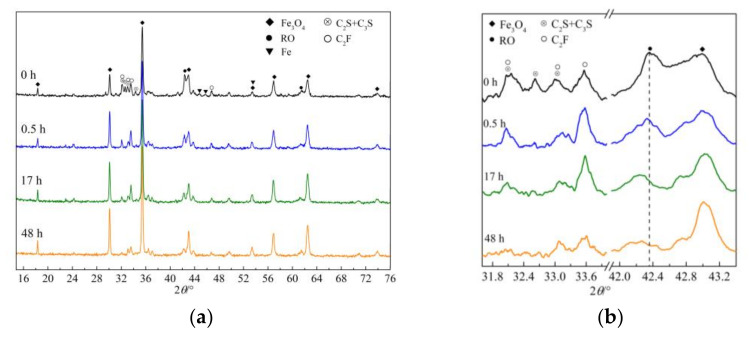
XRD patterns of sample S_1_ and its leaching residue. (**a**) XRD patterns at 15°–76°; (**b**) Magnified XRD patterns.

The XRD patterns of leaching residues of sample S_1_ (leached for 0 h) collected at four time intervals are shown in Figure 4. Specifically, Figure 4a show the XRD patterns of the sample collected at 15–76°, while Figure 4b show a magnified portion of the XRD patterns to highlight the peak position or shape changes of several minerals during leaching. Comparison of the XRD peak intensities of several minerals in the four leaching residues shown in Figure 4a yielded the same qualitative conclusions obtained in the SEM-BEI petrographic analysis: the contents of inert minerals increased, whereas the contents of silicate minerals and C_2_F decrease during leaching by acetum. Since Fe_3_O_4_ is insoluble in acetum and its mass remained unchanged, the approximate content of minerals in the leaching residue was expressed as the ratio of the height of the quantitative peak of each mineral to the height of the quantitative peak of Fe_3_O_4_ to obtain the approximate mineral contents of the sample. The diffraction angles of the quantitative peaks of Fe_3_O_4_, calcium silicate, RO phase, and C_2_F were 35.38°, 32.58°, 42.35°, and 33.46°, respectively. The measurement results are shown in Table 3.

The mineral contents of the leaching residue detailed in Table 3 reveal that the three soluble minerals have different leaching speeds. Among the minerals, calcium silicate demonstrated the highest leaching speed, and the leaching rate of this mineral exceeded 80% within 0.5 h. This mineral was nearly completely leached within 17 h. The RO phase has a leaching rate of over 50% at 0.5 h. This mineral showed a high-leaching speed in the early stages of the leaching process and then a lower leaching speed in later stages. The content of C_2_F remained unchanged or only slightly increased after leaching for 0.5 h. At this time point, the leaching rate of calcium silicate exceeded 80%. The content of C_2_F remarkably decreased after leaching for 17 h, at which point calcium silicate had basically completely leached out of the residue. These findings indicate that C_2_F underwent obvious leaching only after calcium silicate was almost completely leached from the residue. Moreover, the leaching rate of C_2_F was low in the early stages and high in the later stages of leaching. Fe_3_O_4_ was the only insoluble mineral in the sample. Due to the continuous dissolution and mass reduction of soluble minerals, the content of Fe_3_O_4_ in the sample, as well as its diffraction peak intensity, steadily increased.

Figure 4b also show the leaching sequence of calcium silicate and C_2_F. The single characteristic diffraction peak of calcium silicate (32.58°) basically disappeared after 0.5 h, and the two overlying peaks (32.13° and 32.98°) of calcium silicate and C_2_F weakened significantly after 17 h. These results indicate that calcium silicate was rapidly leached within 0.5 h and that C_2_F does not begin to leach notably until the former is completely leached out of the residue.

As shown in Figure 4b, the peak position of the (200) crystal plane of the RO phase gradually shifts during leaching from 42.35° to 42.33°, 42.22°, and 42.18° with increasing leaching time, that is, toward lower angles, in succession. The RO phase is a continuous solid solution with FeO and MgO as main components. The diffraction angles of the (200) crystalline planes of wustite (FeO) and periclase (MgO) are 41.88° and 42.89°, respectively. The experiments also confirmed that the higher the content of FeO in the RO phase, the smaller the diffraction angle of the crystal plane [17]. The shift in the diffraction angle of the RO phase toward a smaller angle during leaching indicates that the FeO content increases, whereas the MgO content decreases in this phase.

Although XRD can qualitatively indicate mineral contents according to the relative strength of the diffraction peak of the mineral phase, it cannot quantitatively determine the mineral contents of IMP. Moreover, this technique is unable to detect minerals with very low contents, such as metallic Fe. Therefore, chemical phase analysis was conducted to determine the mineral composition of the sample quantitatively.

(3) Chemical phase analysis

The contents of calcium silicate, C_2_F, the RO phase, Fe_3_O_4_, and metallic Fe (M-Fe) in sample S_1_ and its leaching residue were quantitatively determined by chemical phase analysis, as described in Section 2.3.2 [26,27]. The results are shown in Table 4.

Variations in the Fe_3_O_4_, C_2_F, calcium silicate, and RO phase contents of sample S_1_ during leaching were consistent with the XRD results obtained earlier. Moreover, metallic Fe was clearly continuously leached from the samples. The five minerals were divided into two categories according to the general trend of their content variation in the leaching residue. The first category was enriched minerals, including Fe_3_O_4_ and C_2_F, which increased in content as leaching continued. These enriched minerals could be further divided into two subgroups according to whether the increase in their content could be sustained throughout the leaching process. The first subgroup was the persistently enriched mineral Fe_3_O_4_; because this mineral is insoluble in acetum, its content increased continuously throughout the leaching process. The second subgroup is the stage-enriched mineral C_2_F. When calcium silicate was present in the sample at the early stages of leaching, it is basically not leached, or its leaching speed is very low; therefore, its content in the leaching residue increases. In the later stages of leaching, calcium silicate in the sample was basically completely leached from the residues. This mineral began to leach out slowly, so its content declined gradually. The second category is leachable minerals, including the RO phase, metallic Fe, and calcium silicate, which decreased in content as leaching proceeded. These minerals could be further divided into two subgroups according to their leaching speed. The first subgroup was slow-leaching minerals, such as the RO phase and metallic Fe, the contents of which decreased by 1.67% and 0.52%, respectively, within 0.5 h. The contents of these minerals also decreased slowly over the course of leaching. The second subgroup was calcium silicate, which was a fast-leaching mineral. The content of this mineral in the residue was reduced by 13.55% within 0.5 h; that is, it was rapidly leached over a very short time, and its content remained at a very low level.

The leaching characteristics of the mineral components in acetum have not been mentioned in the literature. According to the leaching characteristics of minerals determined in this study, the mineral and TFe contents of leaching residues could be estimated by measuring the mineral content of the IMP only.

As shown in Figure 4b, the leaching rates of certain chemical components of the RO phase vary significantly, unlike those of the general mineral phases. In the following section, the RO phase separated from steel slag, and RO phase synthesized from analytically pure reagents were used to compare differences in the leaching rates of MgO and FeO in this mineral. The common characteristics of the selective leaching of these two chemical components were also obtained.

### 3.2. Leaching of RO Phase Samples

#### 3.2.1. XRD Analysis

**Figure 5:**
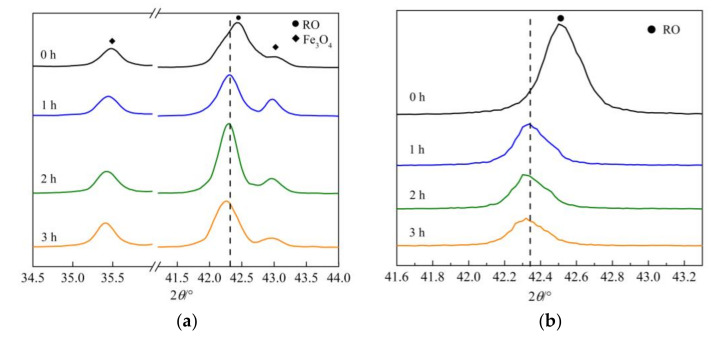
XRD patterns of samples RO_1_ and RO_2_ and their leaching residues. (**a**) RO_1_ and its leaching residues; (**b**) RO_2_ and its leaching residues.

The RO phase content of the two RO phase samples was higher than that in sample S_1_. Thus, the two RO phase samples were selected to obtain results that could better represent the leaching properties of the RO phase. XRD patterns of samples RO_1_ and RO_2_ (leaching time: 0 h) and their leaching residues at three time intervals are shown in Figure 5. The RO phase in sample RO_1_ was the original RO phase generated during steel slag processing, while the RO phase in sample RO_2_ was the RO phase synthesized using analytically pure chemical reagents under laboratory conditions.

As shown in Figure 5a, the peak indicating the (200) crystal plane of the original RO phase shifted toward lower angles from the initial position of 42.44° by 0.13°, 0.15°, and 0.20° after leaching for 1, 2, and 3 h, respectively. In Figure 4b, the peak position of the (200) crystal plane of the original RO phase was 42.35° before leaching and shifted toward lower angles by 0.02°, 0.13°, and 0.17° after leaching for 0.5, 17, and 48 h, respectively. During leaching by acetum, the peak indicating the (200) crystal plane of the two original RO phases gradually shifted to lower angles. In Figure 5b, the peak indicating the (200) crystal plane of the synthesized RO phase was located at 42.50° before leaching and shifted toward lower angles by 0.16°, 0.17°, and 0.18° after leaching for 1, 2, and 3 h, respectively. As described in Section 3.1.2, the RO phase is a continuous solid solution with FeO and MgO as its main chemical components [19], and the peak position of the (200) crystal plane of wustite (FeO) is lower than that of periclase (MgO). The gradual shift of the (200) crystal plane of the RO phase towards lower angles during leaching by acetum indicated that the FeO content gradually increased, whereas the MgO content gradually decreased in this mineral.

As shown in Figure 5a, the diffraction peak of Fe_3_O_4_ in the leaching residues of the RO_1_ sample gradually isolated and appeared at 43.037°; in addition, the diffraction peak intensity of Fe_3_O_4_ at 35.412° gradually increased. This result may be attributed to the decrease in the diffraction peak intensity of the (200) crystal plane of the RO phase. As the peak position shifts toward the left, a space is obtained that could support the appearance of the diffraction peak of Fe_3_O_4_ at 43.037°. However, because the contents of other minerals except Fe_3_O_4_ decreased following leaching, the relative content of Fe_3_O_4_ in the leaching residue and its diffraction peak intensity increased.

After the leaching of the RO phase by acetum, the peaks in its XRD pattern shifted toward lower angles. Thus, the leaching rate of MgO may be inferred to be higher than that of FeO. Since the exact leaching rates of MgO and FeO could not be obtained by XRD, chemical analysis was used to determine the contents of MgO and FeO in the two RO phase samples before and after leaching.

#### 3.2.2. Chemical Analysis

Samples RO_1_ and RO_2_ were leached in acetum for 1, 2, and 3 h to obtain six types of leaching residues and determine their yields. Since the two RO samples could be regarded as two types of leaching residues with a leaching time of 0 h, a total of eight leaching residues were considered in this work. The contents of TFe and MgO in the eight leaching residues were determined, and the TFe contents of the samples were converted to FeO contents. The yields of the leaching residues and the FeO and MgO contents of these residues are in Table 5.

The Fe_3_O_4_, RO phase, C_2_F, and calcium silicate contents of the eight types of leaching residues were determined, and the FeO and MgO contents of the RO phase in each sample were calculated according to the method described in Section 2.3.3. The FeO and MgO contents of the RO phase were marked FeO_RO_ and MgO_RO_, respectively, and the corresponding FeO/MgO molar ratio was calculated from the values of FeO_RO_ and MgO_RO_. These three values are listed in the three columns of the RO phase item in Table 5. The leaching rates of FeO and MgO in the RO phase could be calculated respectively, using the FeO_RO_ and MgO_RO_ values determined in the RO phase sample before leaching, the yield of the leaching residue, and the FeO_RO_ and MgO_RO_ values in the leaching residue. The leaching rates of FeO and MgO in the RO phase are listed in the two columns of the leaching rate item in Table 5.

Analysis of the relationships between the values of FeO_RO_ and MgO_RO_ and the molar ratio of FeO/MgO in the RO phase item in Table 5, in addition to the leaching time, revealed that the FeO content monotonically increased whereas the MgO content monotonically decreased in the two RO phases with increasing leaching time. Thus, the molar ratio of FeO/MgO in the two RO phases monotonically increased with the leaching time. The decrease in MgO content of the original RO phase was larger than that in the synthesized RO phase, and the increase in FeO content of both RO phases was similar. Taking the sample leached for 3 h as an example, the content of MgO in the original RO phase decreased by 13.42%, whereas the content of MgO in the synthesized RO phase decreased by 1.40%. Moreover, the former was 9.6 times that of the latter. By comparison, the content of FeO in the original RO phase increased by 1.81% and the content of Fe in the synthesized RO phase increased by 1.86%.

In Table 5, the leaching rates of MgO and FeO in the two RO phase samples increased monotonically with increasing leaching time, which demonstrated that the two oxides in the RO phase are continuously leached over time without achieving saturation. The leaching rates of MgO and FeO in the original RO phase were higher than those in the synthesized RO phase. For example, the respective leaching rates of MgO and FeO in the original RO phase sample leached for 3 h were 2.3 and 1.7 times higher than those in the synthesized RO phase at the same time point. The leaching rate of MgO was higher than that of FeO in both RO phase samples. In the original RO phase, after leaching for 1, 2, and 3 h, the respective leaching rates of MgO were 1.4, 1.9, and 1.9 times those of FeO. In the synthesized RO phase sample, the respective leaching rates of MgO were 1.3, 1.4, and 1.4 times those of FeO at the same leaching times. The selective leaching characteristics of FeO and MgO in the RO phase have not been reported in the literature.The results collectively indicate a difference in the leaching rates of MgO and FeO in the RO phase, and the difference in these rates is more significant in the original RO phase sample. Therefore, acetum leaching could be adopted to enrich MgO in the leaching solution and FeO in the leaching residue.

SEM-EDS was applied to analyze the chemical compositions of the two RO phase samples and their leaching residues after leaching for 2 h (labeled RO_1_-2 and RO_2_-2) and demonstrated the reliability of the chemical analysis results. The results are shown in Table 6. After RO_1_ was leached, the contents of Mg, Ca, Si, and Al decreased, whereas the content of Fe increased in the leaching residue. The molar ratio of FeO/MgO also increased. After the RO_2_ sample was leached, the content of Fe increased, whereas the content of Mg decreased in the RO phase. After leaching, the Fe content in both RO phase samples increased, whereas the contents of Mg and other components decreased. These findings are consistent with the chemical analysis results.

## 4. Conclusions

In this paper, the IMP and two RO samples were selected to study the leaching chemical and mineral components of leaching residues during the acetum leaching. In addition, the leaching characteristics of chemical and mineral components in IMP and the leaching rates of MgO and FeO in the RO phase were studied. The following conclusions were made:

(1) CaO and SiO_2_ had high-leaching rates; indeed, the respective leaching rates of these components were 3.9 and 3.3 times higher than that of Fe. MgO has a moderate leaching rate of approximately 3.0 times that of Fe. Fe had the lowest leaching rate among these components. CaO and SiO_2_ had high-leaching speeds and were mostly leached out within 0.5 h. By comparison, MgO and Fe had lower leaching speeds and could be leached out after 22 h.
(2) Fe_3_O_4_ was insoluble in acetum, and C_2_F began to leach slowly in small amounts only after calcium silicate was completely leached. The RO phase and metallic Fe had low-leaching speeds and slow-leaching rates. Calcium silicate can be mostly leached within 0.5 h, and its leaching rate is fairly high.
(3) The TFe content increased greatly, the SiO_2_ content decreased slightly, and the CaO and MgO contents decreased greatly in the leaching residue. Moreover, the Fe_3_O_4_ content increased continuously, the C_2_F content first increased and then decreased, the RO phase and metallic Fe contents decreased slowly, and the calcium silicate content decreased significantly within 0.5 h.
(4) MgO and FeO in the RO phase were continuously leached over time, and the maximum leaching rate of MgO was 1.9 times that of FeO. The MgO content decreased, whereas the FeO content increased during the leaching of the RO phase. The leaching rates of MgO and FeO in the original RO phase were higher than those in the synthesized RO phase, and the difference between these rates was quite large.
(5) The results of the present study lay a theoretical foundation for future research and development efforts to enhance acetum leaching technologies to improve the TFe content of IMP.

## Figures and Tables

**Table 1:** Chemical compositions of the tested materials determined by XRF analyses/%.

Sample	Fe_2_O_3_	MgO	CaO	SiO_2_	Al_2_O_3_	MnO	P_2_O_5_	TiO_2_	Cr_2_O_3_	SO_3_	V_2_O_5_	Total
S_1_	51.06	16.00	17.66	7.59	1.90	3.54	1.30	0.40	0.27	0.18	0.09	99.98
RO_1_	54.27	18.30	9.44	5.51	3.95	4.15	2.63	1.07	0.30	0.16	0.16	99.93

All “%” refers to the percentage of mass.

**Table 2:** Chemical compositions of samples S_1_ and S_1_-0.5/%.

Sample	O	Fe	Ca	Mg	Si	Al
S_1_	60.95	14.93	9.63	9.08	3.48	1.94
S_1_-0.5	58.05	29.05	2.44	6.82	2.80	0.84

**Table 3:** Ratios of the XRD quantitative peak heights of three minerals to Fe_3_O_4_.

Time/h	C_2_S + C_3_S	RO Phase	C_2_F
0	0.15	0.33	0.20
0.5	0.03	0.16	0.21
17	0.01	0.10	0.18
48	0.00	0.08	0.09

**Table 4:** Mineral phase contents of sample S_1_ and its leaching residue/%.

Time/h	Fe_3_O_4_	C_2_F	C_2_S + C_3_S	RO Phase	M-Fe
0	43.27	11.03	16.04	24.14	1.59
0.5	59.14	11.97	2.49	22.47	1.07
17	67.39	13.43	1.34	14.32	0.98
48	77.30	9.85	0.98	7.86	0.14

**Table 5:** Yield and FeO and MgO contents of the leaching residues and the FeO and MgO contents and their corresponding leaching rates in the RO phase.

Sample	Time/h	Leaching Residue			RO Phase			Leaching Rate	
		Yield /%	FeO /%	MgO /%	FeO_RO_ /%	MgO_RO_ /%	FeO/MgO	FeO_RO_ /%	MgO_RO_ /%
RO_1_	0	-	57.87	24.04	46.11	52.49	0.49	-	-
	1	79.7	66.27	22.14	47.66	49.32	0.54	17.6	25.1
	2	76.7	66.85	20.33	47.82	42.18	0.63	20.5	38.4
	3	73.5	67.12	19.34	47.92	39.07	0.68	23.6	45.3
RO_2_	0	-	-	-	64.59	36.26	1.00	-	-
	1	91.7	-	-	65.88	36.10	1.02	6.5	8.7
	2	88.7	-	-	65.92	35.38	1.05	10.2	14.2
	3	83.7	-	-	66.45	34.86	1.07	13.9	19.6

**Table 6:** Chemical compositions of the two RO phase samples before and after leaching/%.

Sample	O	Fe	Ca	Mg	Si	Al	FeO/MgO
RO_1_	62.95	15.69	10.47	5.71	2.24	2.94	1.50
RO_1_-2	62.48	23.55	8.86	2.77	1.38	0.96	2.66
RO_2_	47.01	27.55	25.44	25.44	-	-	1.08
RO_2_-2	46.26	30.25	23.49	23.49	-	-	1.29

## Data Availability

The data presented in this study are available on request from the corresponding author.
